# Supplementary material for: Germ granule localization of nematode Argonaute WAGO-4 ensures fidelity in small RNA loading
Source: EMBO J. 2025 Oct 22;44(23):7211–41. doi: 10.1038/s44318-025-00606-x (PMC12669755; doi:10.1038/s44318-025-00606-x)
Supplement: Supplementary file 11 — Expanded View Figures [file 44318_2025_606_MOESM11_ESM.pdf]

Expanded View Figures

Figure EV1. Effect of FG mutations on GLH protein function and reproductive outcomes.

(**A**) Table representing the number of FG dipeptides in GLH proteins: total number of FG dipeptides outside of helicase domains and outside of helicase and zinc finger (ZF) regions are shown. All FG dipeptides outside of helicase and ZF regions (second column) were mutated. (**B**) Graph representing the number of progeny for worms of the indicated genotypes grown at 20 °C. Each dot represents the progeny count of a single worm ($n = 17$–20). Horizontal lines represent mean values, and error bars indicate SD. Statistical analysis: Kruskal–Wallis test; the corresponding *P* values are reported in Dataset EV2. Groups sharing at least one letter are statistically indistinguishable ($P < 0.05$). (**C**) Graph representing the number of progeny for worms of the indicated genotypes grown at 26 °C for one generation. This is the second replicate of the experiment shown in Fig. 1B, which exhibited a more severe phenotype. Each dot represents the progeny count of a single worm ($n = 16$–18). Horizontal lines represent mean values, and error bars indicate SD. Statistical analysis: the Kruskal–Wallis test; the corresponding *P* values are reported in Dataset EV2. Groups sharing at least one letter are statistically indistinguishable ($P < 0.05$). (**D**) Graph representing the number of progeny for worms of the indicated genotypes grown at 25 °C. Each dot represents the progeny count of a single worm ($n = 18$–20). Horizontal lines represent mean values, and error bars indicate SD. Statistical analysis: Kruskal–Wallis test; the corresponding *P* values are reported in Dataset EV2. Groups sharing at least one letter are statistically indistinguishable ($P < 0.05$). (**E**) Graph representing the number of progeny for worms of the indicated genotypes (single *glh* mutants: *glh-1(F→A), glh-2(ΔFG)* and *glh-4(F→A)*) grown at 26 °C. Each dot represents the progeny count of a single worm ($n = 18$–19). Horizontal lines represent mean values, and error bars indicate SD. Statistical analysis: Kruskal–Wallis test; the corresponding *P* values are reported in Dataset EV2. Groups sharing at least one letter are statistically indistinguishable ($P < 0.05$). (**F**) Schematic representation of wild-type (WT) and mutated form DDX-19. Exon 4 which harbors most FG repeats of DDX-19 was deleted in the native locus. 4×FG mutant contains this mutation together with FG mutations in GLH-1, GLH-2, and GLH-4 proteins, as described in Fig. 1A. (**G**) Graph representing the number of progeny for worms of the indicated genotypes grown at 26 °C for one generation. Each dot represents the progeny count of a single worm ($n = 17$–18). Horizontal lines represent mean values, and error bars indicate SD. Statistical analysis: the Kruskal–Wallis test; the corresponding *P* values are reported in Dataset EV2. Groups sharing at least one letter are statistically indistinguishable ($P < 0.05$). (**H**) Graph showing relative absorbance of reactions containing GLH-1 or GLH-1(F→A) with or without RNA, normalized to a blank reaction. Reactions were prepared using the EnzCheck Phosphate Assay Kit (Thermo Fisher). Average normalized absorbance over time ($n = 3$) indicating the release of inorganic phosphate is plotted. Error bars indicate SD. (**I**) Micrographs of early embryos from WT worms (upper) and an unfertilized oocyte from 3×FG mutant worms (lower) grown at 26 °C for one generation. Samples were stained with FM-64 (plasma membrane) and DAPI (DNA). Properly formed eggshells in WT embryos (black arrow) prevent staining by FM-64 and DAPI, whereas unfertilized oocytes lack a complete eggshell, so plasma membrane (white arrows) and DNA are stained. Scale bar: 20 µm. (**J**) Quantification of unfertilized oocytes in worms grown at 20 °C. Each worm was categorized into one of three groups: (1) containing exclusively embryos, (2) containing at least one embryo and at least one unfertilized oocyte, or (3) containing exclusively unfertilized oocytes (group 3 was not observed). Data represent averages from three independent experiments ($n = 50$ worms/experiment), with error bars indicating SD. (**K**) Quantification of unfertilized oocytes in uteri of worms grown at 26 °C for one generation. Each worm was categorized into one of three groups: (1) containing exclusively embryos, (2) containing at least one embryo and at least one unfertilized oocyte, or (3) containing exclusively unfertilized oocytes. Data represent the average proportion of worms in each category from three independent experiments ($n = 48.3 \pm 3.1$ worms per experiment), with error bars indicating SD. (**L**) Graph representing the number of progeny for worms of the indicated genotypes grown at 26 °C for one generation and mated with WT males expressing PGL-3::mCherry. Each dot represents the progeny count of a single worm ($n = 8$–10). Horizontal lines represent mean values, and error bars indicate SD. Statistical analysis: the Kruskal–Wallis test; the corresponding *P* values are reported in Dataset EV2. Groups sharing at least one letter are statistically indistinguishable ($P < 0.05$).

© The Author(s)

A

Protein	# FG dipeptides outside helicase region	# FG dipeptides outside helicase and ZF regions
GLH-1	19	15
GLH-2	32	24
GLH-4	43	43

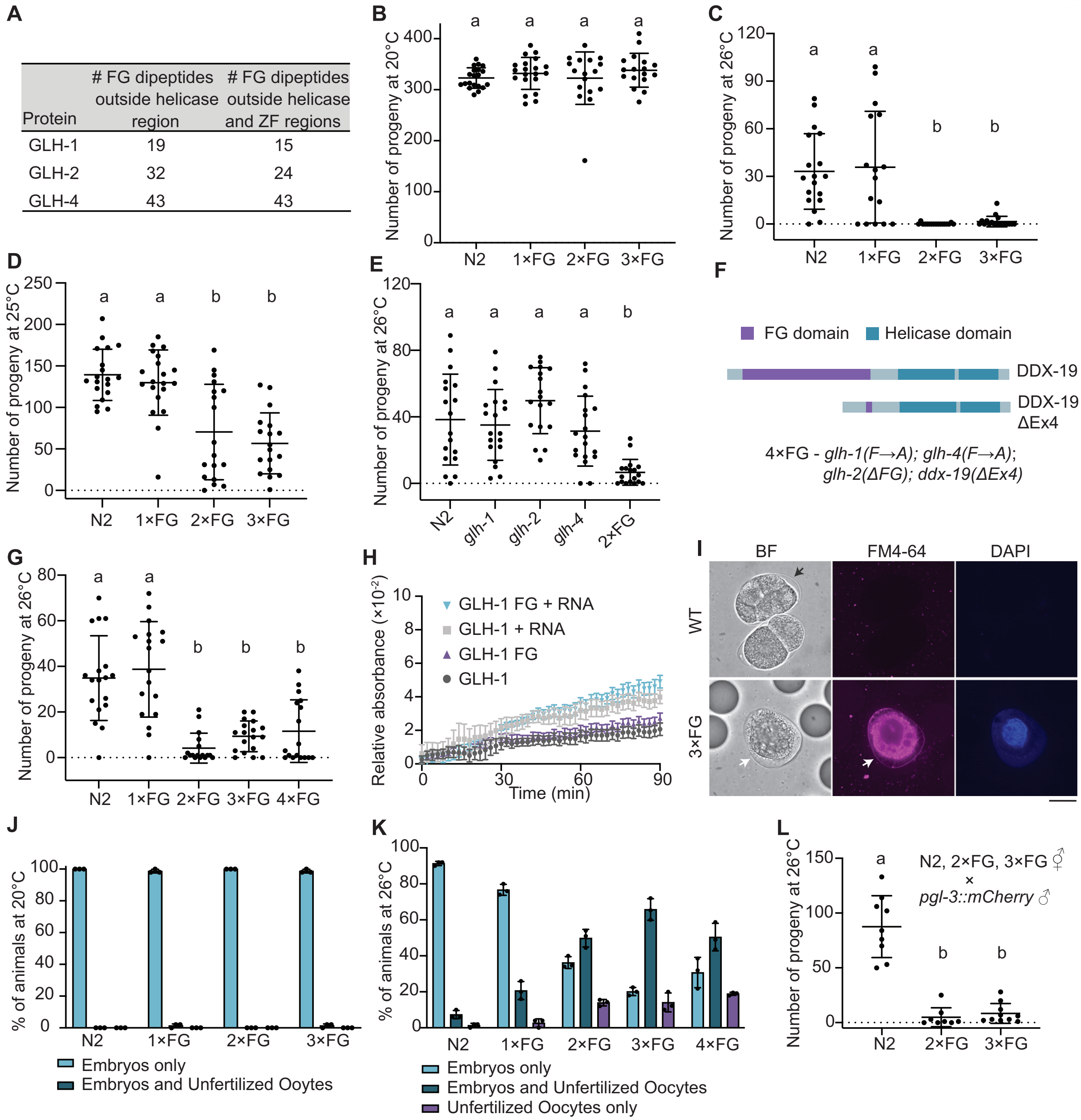

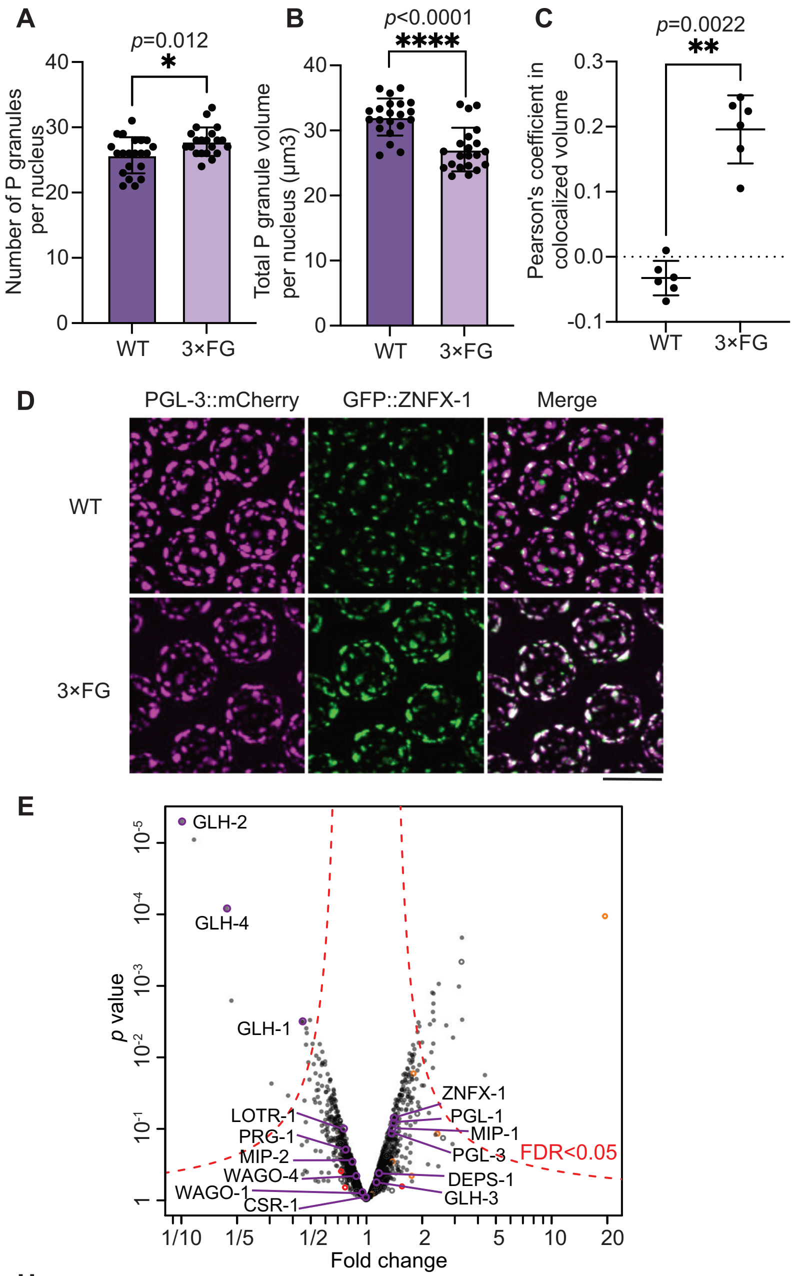

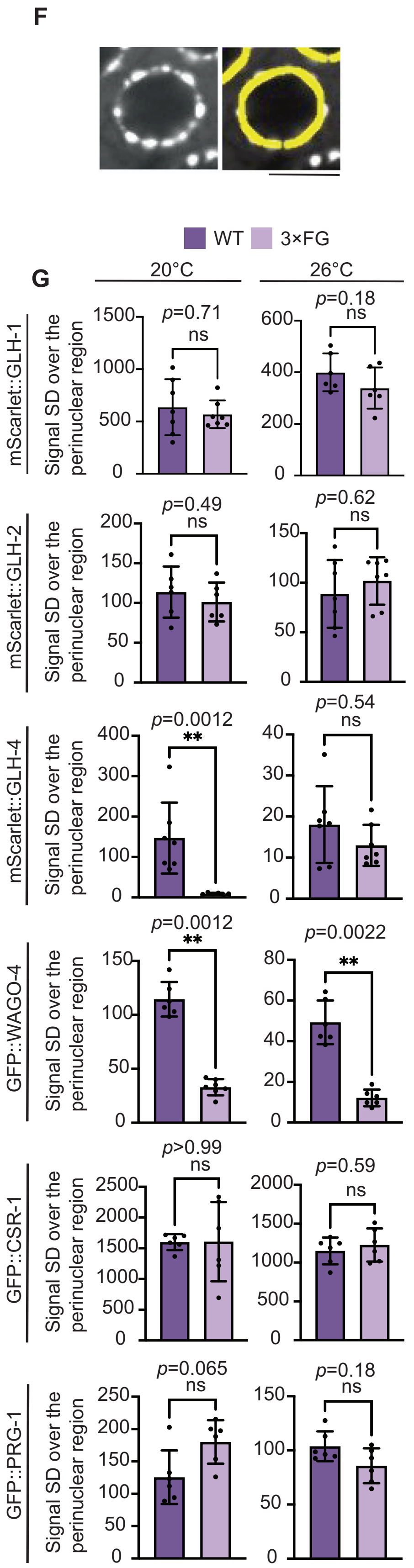

H

20°C	Fold change	Adjusted *p* value	FDR <0.01
GLH-1	1.08	0.84	false
GLH-2	1.31	0.63	false
GLH-4	1.68	0.04	false
WAGO-4	1.24	0.29	false

26°C	Fold change	Adjusted *p* value	FDR <0.01
GLH-1	1.02	0.78	false
GLH-2	1.71	0.03	false
GLH-4	1.27	0.04	false
WAGO-4	0.79	0.03	false

Protein expression fold enrichment 3×FG vs. WT, determined by MS

© The Author(s)

Figure EV2. Impact of GLH-FG mutations on germ granule localization and composition.

(**A**) Graph showing the average number of P granules per nucleus in WT and 3×FG mutant worms. Data were obtained from 3D representations of pachytene nuclei in live worms expressing PGL-3::mCherry at 20 °C. Each dot represents the number of P granules in a single nucleus ($n = 21$). Bars indicate mean values, and error bars represent SD. For statistical analysis, two-tailed Mann–Whitney test was performed, *P* value indicated in the graph. (**B**) Graph showing the total P granule volume per nucleus (μm^3) in WT and 3×FG mutant worms. Data were calculated from 3D representations of pachytene nuclei in live worms expressing PGL-3::mCherry at 20 °C. Each dot represents the total P granule volume of a single nucleus ($n = 21$). Bars indicate mean values, and error bars represent SD. For statistical analysis, two-tailed Mann–Whitney test was performed, *P* value indicated in the graph. (**C**) Graph showing Pearson's coefficient for colocalized volume between PGL-3::mCherry and GFP::ZNFX-1 in WT and 3×FG mutant worms. Each dot represents the coefficient for a single worm, based on approximately 30 pachytene nuclei per image ($n = 6$). Horizontal lines represent mean values, and error bars indicate SD. For statistical analysis, two-tailed Mann–Whitney test was performed, *P* value indicated in the graph. (**D**) Fluorescence micrographs showing maximum intensity projections of pachytene nuclei in live worms co-expressing PGL-3::mCherry and GFP::ZNFX-1 in WT (upper) and 3×FG mutant (lower) backgrounds, grown at 20 °C. Scale bar: 5 µm. (**E**) Volcano plot showing fold enrichment of proteins after streptavidin pulldown in PGL-3::TurboID expressing worms grown at 20 °C (3×FG vs. WT), determined by mass spectrometry ($n = 3$ biological replicates). Statistical significance was calculated using limma (Ritchie et al, 2015). Identified known P granule and Z granule proteins are highlighted. (**F**) Representative fluorescent micrographs showing a single germline nucleus in a live worm expressing a fluorescently labeled germ granule protein (left panel) and the same nucleus with the ROI over the perinuclear region indicated as a yellow line (right panel). This is a representation of the ROIs used for calculating signal standard deviation in Fig. EV2G. Scale bar: 5 µm. (**G**) Graphs showing the average signal standard deviation over the perinuclear region in live WT and 3×FG mutant worms expressing a single fluorescently tagged protein (from top to bottom): mScarlet::GLH-1, mScarlet::GLH-2, mScarlet::GLH-4, GFP::WAGO-4, GFP::CSR-1, and GFP::PRG-1. Quantification was performed on a single confocal slice representing the middle plane of the analyzed nucleus, as shown in Fig. EV2F (each image contained multiple pachytene nuclei). Worms were grown at 20 °C (left) or 26 °C (right). Each dot represents the average signal standard deviation for a single worm ($n = 6$), bars indicate mean values, and error bars represent SD. For statistical analysis, two-tailed Mann–Whitney test was performed, *P* values indicated in the graph. (**H**) Protein levels analysis by mass spectrometry of GLH proteins and WAGO-4. Mass spectrometry was performed on worm lysates from animals grown at either 20 °C or 26 °C ($n = 3$ biological replicates). Fold changes (3×FG mutant relative to WT), corresponding adjusted *P* values and a true/false indicator for whether the false discovery rate (FDR) is below 0.01 (as determined by limma) are shown for each protein.

© The Author(s)

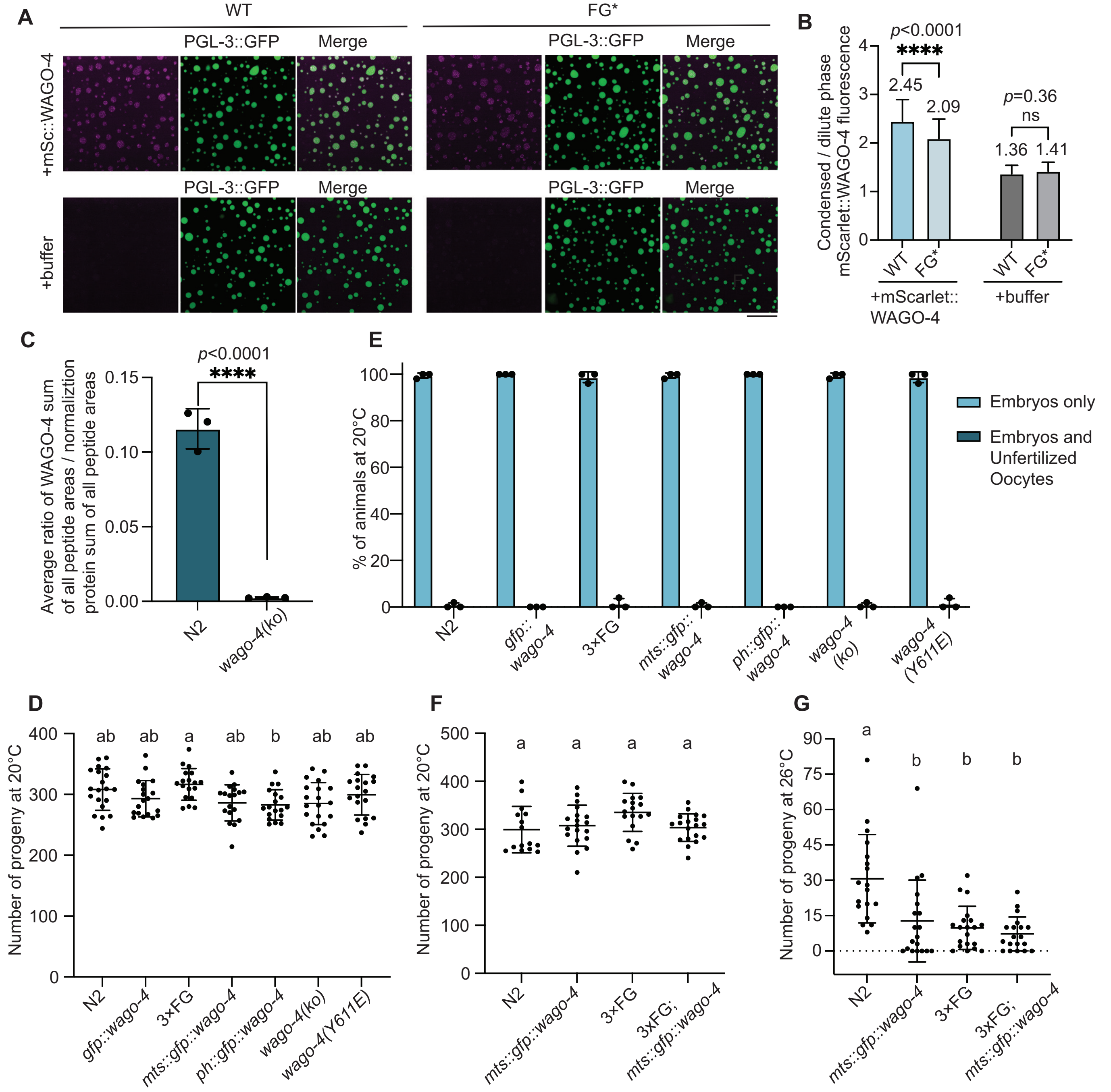
A
WT
FG*
PGL-3::GFP
Merge
+mSc::WAGO-4
+buffer
B
p<0.0001
****
2.45
2.09
p=0.36
ns
1.36 1.41
Condensed / dilute phase mScarlet::WAGO-4 fluorescence
WT FG* WT FG*
+mScarlet:: WAGO-4
+buffer
C
p<0.0001
****
Average ratio of WAGO-4 sum of all peptide areas / normaliztion protein sum of all peptide areas
N2
wago-4(ko)
E
% of animals at 20°C
Embryos only
Embryos and Unfertilized Oocytes
N2
gfp:: wago-4
3×FG
mts::gfp:: wago-4
ph::gfp:: wago-4
wago-4 (ko)
wago-4 (Y611E)
D
ab ab a ab b ab ab
Number of progeny at 20°C
N2
gfp::wago-4
3×FG
mts::gfp::wago-4
ph::gfp::wago-4
wago-4(ko)
wago-4(Y611E)
F
a a a a
Number of progeny at 20°C
N2
mts::gfp::wago-4
3×FG
3xFG; mts::gfp::wago-4
G
a b b b
Number of progeny at 26°C
N2
mts::gfp::wago-4
3×FG
3xFG; mts::gfp::wago-4

 © The Author(s)

Figure EV3. WAGO-4 mislocalization from germ granules and its contribution to the 3×FG phenotype.

(**A**) Fluorescent micrographs of condensates containing PGL-1, PGL-3 (5% PGL-3::mEGFP), GLH-1, and GLH-4 with the addition of mScarlet::WAGO-4 (upper row) or protein storage buffer (lower row). Condensates contained WT GLH-1 and GLH-4 (left) or F → A mutant GLH-1 and GLH-4 (right, FG*). Each image shows a single confocal plane. Scale bar: 40 µm. (**B**) Second replicate of the experiment shown in Fig. 3D. Graph showing the ratio of mScarlet::WAGO-4 signal intensity in the condensed phase vs. the dilute phase for WT and FG mutant condensates. Six ROIs were defined per image, and six images were analyzed for each condition ($n = 36$). Bars represent average values with SD. Numbers above bars indicate average values. Statistical analysis was performed using a two-tailed Mann–Whitney test, P values indicated in the graph. mScarlet::WAGO-4 storage buffer was used as a negative control, and ratios were calculated in the same way. (**C**) Graph showing results from targeted PRM-MS analysis of WAGO-4 protein levels in *wago-4(ko)* strain where a stop codon was introduced into the second exon of *wago-4*. The average ratio of the sum of all WAGO-4 peptide areas vs. the sum of all peptide areas for five normalization proteins (AHCY-1, RPL-36, RPL-22, EFT-3, and RPL-27) is shown. N2 was used as a WT control. Statistical analysis was performed using a one-tailed Welch's t test on $\log_2$-transformed intensity ratios, P value indicated in the graph ($n = 3$ biological replicates). (**D**) Graph representing the number of progeny for worms of the indicated genotypes grown at 20 °C in a single experiment. Each dot represents the progeny count of a single worm ($n = 18$–20). Horizontal lines represent mean values, and error bars indicate SD. Statistical analysis: the Kruskal–Wallis test; the corresponding P values are reported in Dataset EV2. Groups sharing at least one letter are statistically indistinguishable ($P < 0.05$). (**E**) Quantification of unfertilized oocytes in worms grown at 20 °C. Each worm was categorized into one of three groups: (1) containing exclusively embryos, (2) containing at least one embryo and at least one unfertilized oocyte, or (3) containing exclusively unfertilized oocytes (group 3 was not observed in this experiment). Data represent averages from three independent experiments ($n = 50$ worms/experiment), with SD indicated by error bars. (**F**) Graph representing the number of progeny for worms of the indicated genotypes grown at 20 °C. Each dot represents the progeny count of a single worm ($n = 15$–19). Horizontal lines represent mean values, and error bars indicate SD. Statistical analysis: Kruskal–Wallis test; the corresponding P values are reported in Dataset EV2. Groups sharing at least one letter are statistically indistinguishable ($P < 0.05$). (**G**) Graph representing the number of progeny for worms of the indicated genotypes grown at 26 °C. Each dot represents the progeny count of a single worm ($n = 18$–20). Horizontal lines represent mean values, and error bars indicate SD. Statistical analysis: Kruskal–Wallis test; the corresponding P values are reported in Dataset EV2. Groups sharing at least one letter are statistically indistinguishable ($P < 0.05$).

© The Author(s)

A

	20°C		26°C	
Regulation vs. WT	Up	Down	Up	Down
3×FG	634	831	225	218
wago-4(Y611E)	381	302	152	233
wago-4(ko)	737	735	124	310

B

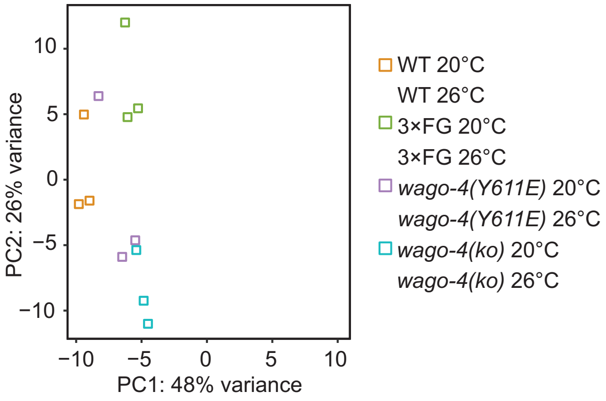

C

Histone/gene	hil-5	his-1	his-16	his-17	his-18	his-2	his-3	his-4	his-40	**his-48**	his-5	**his-59**	his-60	**his-61**	**his-62**	**his-63**	his-64	his-67	his-68	his-7	**his-72**	his-74
H1	•																					
H2A			•				•							•					•	•		
H2B								•														
H3				•		•			•			•				•						
H4		•			•						•		•				•	•				
putative H2B										•					•							
H3.3																					•	•

D

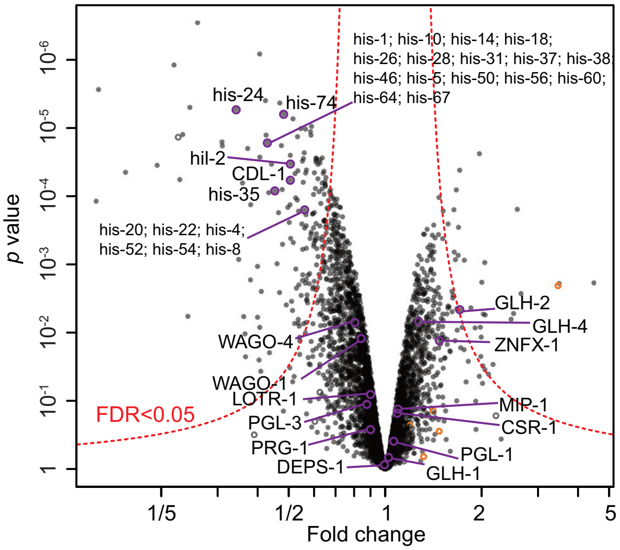

Figure EV4. Temperature-dependent transcriptomic changes in GLH-FG and WAGO-4 mutants and histone gene downregulation in 3×FG mutants.

(**A**) Table showing the number of up- and downregulated genes in 3×FG, *wago-4(Y611E)*, and *wago-4(ko)* worms grown at 20 °C or 26 °C ($p < 0.05$). (**B**) PCA plot based on the top 500 most variable genes. Three biological replicates were analyzed for each genotype and temperature condition, with each replicate individually plotted. The legend on the right indicates the symbol for each condition. (**C**) Table listing all histone genes downregulated in 3×FG vs. WT based on mRNA sequencing at 20 °C. Each column represents a histone gene, and rows indicate the histone type it encodes. Genes highlighted in bold also showed increase in WAGO-4-associated 22G-RNAs in 3×FG compared to WT (Fig. EV5C). (**D**) Volcano plot showing fold enrichment of proteins from total worm lysates of worms grown at 26 °C (3×FG vs. WT), determined by mass spectrometry ($n = 3$ biological replicates). Statistical significance was calculated using limma (Ritchie et al, 2015). Detected histone proteins or clusters and known germ granule proteins are highlighted.

© The Author(s)

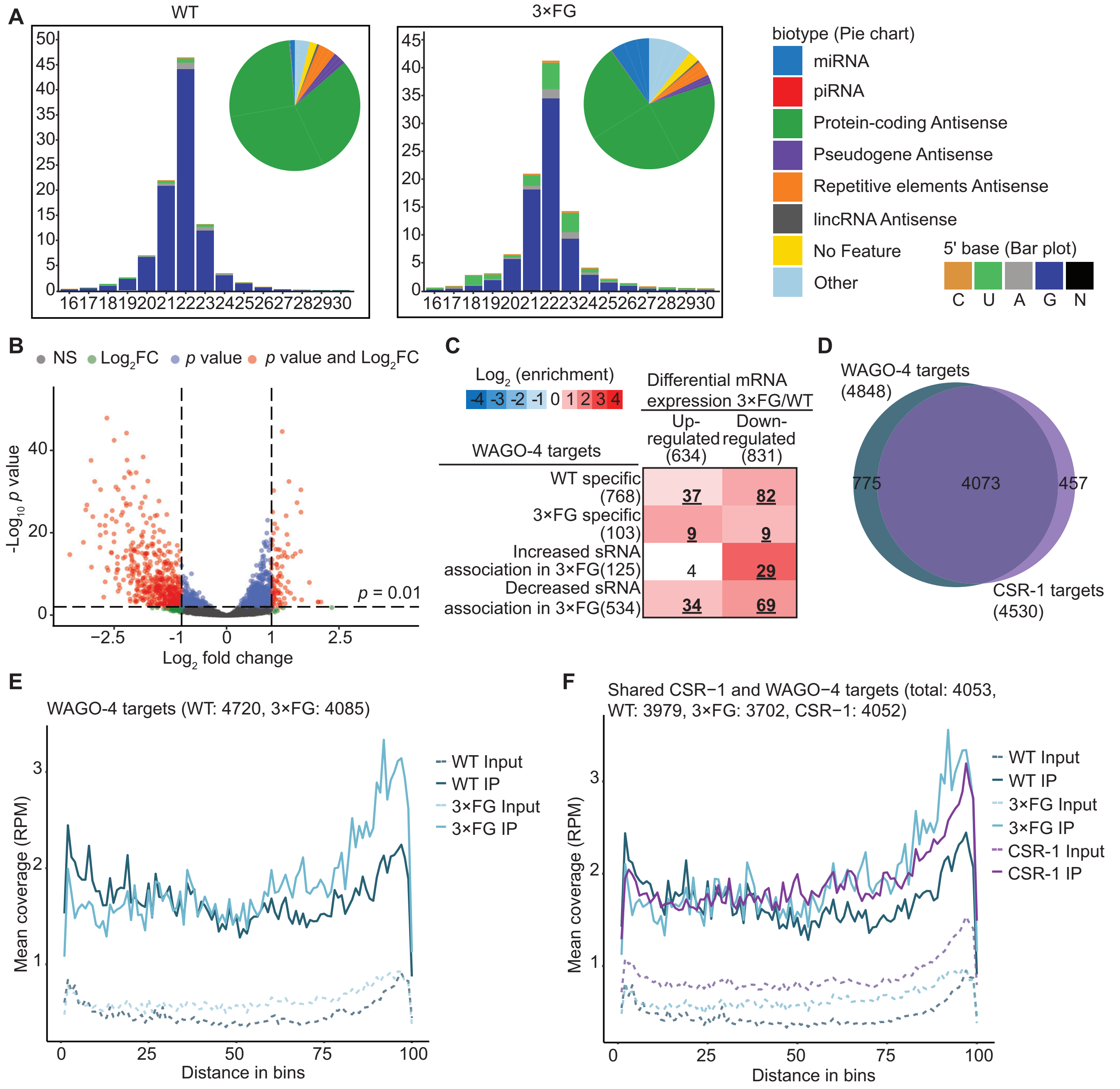
A
WT
3×FG
161718192021222324252627282930
biotype (Pie chart)
miRNA
piRNA
Protein-coding Antisense
Pseudogene Antisense
Repetitive elements Antisense
lincRNA Antisense
No Feature
Other
5' base (Bar plot)
C U A G N
B
NS Log2FC p value p value and Log2FC
-Log10 p value
p = 0.01
Log2 fold change
C
Log2 (enrichment)
-4 -3 -2 -1 0 1 2 3 4
Differential mRNA expression 3×FG/WT
Up-regulated (634)
Down-regulated (831)
WAGO-4 targets
WT specific (768) 37 82
3×FG specific (103) 9 9
Increased sRNA association in 3×FG(125) 4 29
Decreased sRNA association in 3×FG(534) 34 69
D
WAGO-4 targets (4848)
775
4073
457
CSR-1 targets (4530)
E
WAGO-4 targets (WT: 4720, 3×FG: 4085)
Mean coverage (RPM)
Distance in bins
WT Input
WT IP
3×FG Input
3×FG IP
F
Shared CSR−1 and WAGO−4 targets (total: 4053, WT: 3979, 3×FG: 3702, CSR−1: 4052)
CSR-1 Input
CSR-1 IP

Figure EV5. Analysis of WAGO-4–associated 22 G RNAs and target specificity in 3×FG mutants.

(**A**) Bar plots showing the 5′ nucleotide composition and length distribution of sRNAs in each Argonaute IP. Pie charts depict the proportion of sRNAs corresponding to each genetic element (biotype). Data represent the average of three biological replicates. IPs were performed using 3xFLAG::GFP::WAGO-4 in young adult worms. (**B**) Volcano plot showing Log_2 fold enrichment of 22G-RNAs targeting WAGO-4 targets in 3×FG vs. WT worms grown at 20 °C. Each dot represents the average Log_2 fold change ($n = 3$ biological replicates). Differential expression and statistical analysis were performed using DESeq2 (Love et al, 2014). Targets with an average Log_2 fold change of <−1 or >1 and $P < 0.01$ were considered to have a significant decrease or increase in WAGO-4-associated 22G-RNAs, respectively (indicated by red dots). (**C**) Enrichment of WAGO-4 sRNA targets in four groups: WT-specific targets, 3×FG-specific targets, targets with a decrease in WAGO-4-associated sRNAs in 3×FG, and targets with an increase in WAGO-4-associated sRNAs in 3×FG. These groups were compared to genes up- or downregulated in 3×FG/WT based on mRNA sequencing. All data represent worms grown at 20 °C. Log_2 enrichment is indicated by color according to the scale shown, and significant enrichment or depletion ($P < 0.05$, Fisher's exact test) is highlighted in bold and underlined. (**D**) Venn diagram showing the overlap of WAGO-4 targets in WT worms (this study) with CSR-1 targets (Seroussi et al, 2023). Both datasets were derived from sRNA sequencing after Ago IP from adult worms grown at 20 °C. Numbers in brackets indicate the size of each group. (**E**) Metagene profiles for sRNAs complementary to protein-coding gene WAGO-4 targets (all identified targets in WT and 3×FG worms grown at 20 °C). Size-normalized targets were partitioned into 100 bins, and the mean coverage (RPM) for each bin was plotted. Solid lines represent IP values, and dashed lines represent input values. The number of identified targets for each genotype is indicated in brackets. (**F**) Metagene profiles for sRNAs complementary to protein-coding gene WAGO-4 and CSR-1 targets. CSR-1 targets were identified from CSR-1 IP in WT worms (Seroussi et al, 2023). Size-normalized targets were partitioned into 100 bins, and mean coverage (RPM) for each bin was plotted. Solid lines represent IP values, and dashed lines represent input values. The total number of genes analyzed was determined as WAGO-4 and CSR-1 shared targets based on published data (Seroussi et al, 2023), and the number of identified targets for each genotype is indicated in brackets.

 © The Author(s)

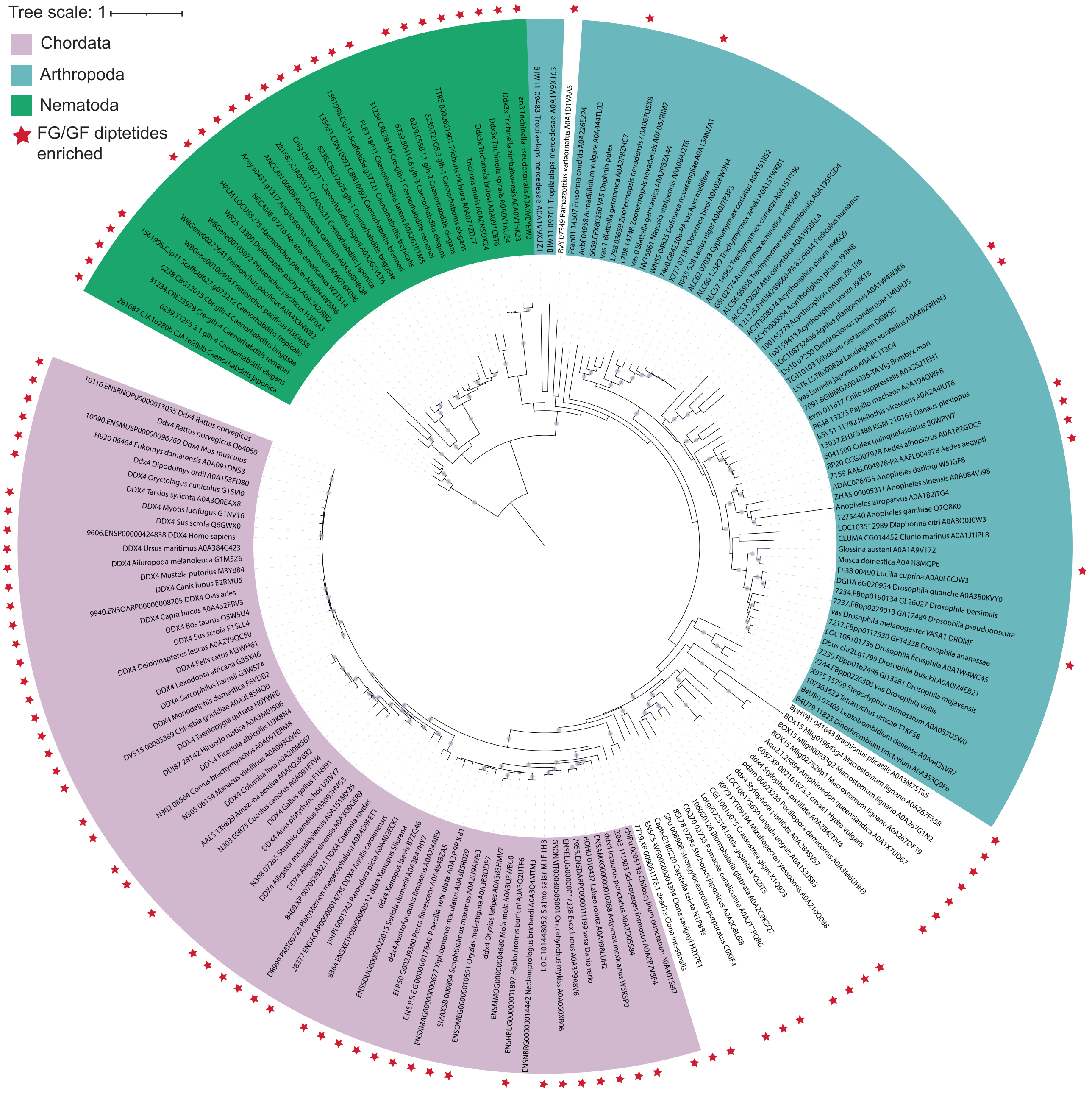

Figure EV6. Phylogenetic tree of Vasa orthologs.

Branches that are supported by an ultrafast bootstrap (UFBoot) value >=95% are indicated by a gray dot. Branch lengths represent the inferred number of amino acid substitutions per site, and branch labels are composed of gene name (if available), genus, species, and accession number. Those proteins which have significant enrichment of FG or GF dipeptides enriched in their sequence (excluding the helicase domains) are highlighted with red stars ($P < 0.005$).

© The Author(s)
